# Association between High-Density Lipoprotein Cholesterol to Apolipoprotein A-I Ratio and Nonalcoholic Fatty Liver Disease: A Cross-Sectional Study

**DOI:** 10.1155/2021/6676526

**Published:** 2021-06-04

**Authors:** Hangkai Huang, Jinghua Wang, Lei Xu, Min Miao, Chengfu Xu

**Affiliations:** ^1^Department of Gastroenterology, The First Affiliated Hospital, Zhejiang University School of Medicine, Hangzhou 310003, China; ^2^Department of Gastroenterology, Zhejiang University Ningbo Hospital, Ningbo 315010, China; ^3^Department of Internal Medicine, Zhenhai Lianhua Hospital, Ningbo 315207, China

## Abstract

**Background:**

This study aimed to explore the association between high-density lipoprotein cholesterol to apolipoprotein A-I ratio (HDL-C/apo A-I) and nonalcoholic fatty liver disease (NAFLD).

**Methods:**

A total of 9025 Chinese adults were enrolled in this cross-sectional study, who presented their annual health checkups at Zhenhai Lianhua Hospital, Ningbo, during 2017.

**Results:**

The NAFLD prevalence was 33.7%, and HDL-C/apo A-I was significantly decreased in NAFLD patients, as well as in lean NAFLD and in patients with NAFLD-related advanced fibrosis (all *P* < 0.001). The prevalence of NAFLD and components of metabolic syndrome are inversely associated with HDL-C/apo A-I (*P* < 0.001). Multivariate logistic regression analysis show that HDL-C/apo A-I is inversely associated with the risk of NAFLD (odds ratio: 0.353, 95% confidence interval: 0.257–0.486; *P* < 0.001).

**Conclusions:**

Our results suggested that increased HDL-C/apo A-I is significantly associated with a decreased risk of NAFLD.

## 1. Introduction

Nonalcoholic fatty liver disease (NAFLD) is featured by excessive accumulation of lipid within hepatocytes without excess alcohol consumption and other causes of hepatic steatosis [1]. NAFLD is reported to affect about 25% of the world's population [2], and the most recent prevalence of NAFLD is as high as 29.2% in China [3]. NAFLD is divided into two subtypes as nonalcoholic fatty liver (NAFL) and nonalcoholic steatohepatitis (NASH). NAFL shows usually a benign nature. However, NASH is the progressive subtype of NAFLD and has an increased potential to progress to fibrosis, cirrhosis, and hepatocellular carcinoma [4]. Dulai et al. [5] observed that advanced fibrosis is positively associated with increased hepatic-related and all-cause mortality in NAFLD patients. Therefore, great emphasis should be placed on early identification of hepatic fibrosis in the progression of NAFLD.

The increasing prevalence of NAFLD is accompanying the increasing prevalence of type 2 diabetes mellitus (T2DM), obesity, and other components of metabolic syndrome (MetS) [6]. However, NAFLD is also commonly occurred in nonobese individuals [7]. Zou et al. [8] observed that nonobese NAFLD makes up 22.7% of the NAFLD in the United States, analyzed by using the 1999–2016 NHANES databases. Our previous study found that nonobese NAFLD prevalence was 7.27% in China, and 8.88% nonobese developed NAFLD during a medium of five years of follow-up [9]. Thus, great importance should be attached to the risk factors related to NAFLD in nonobese populations.

In NAFLD patients, atherogenic dyslipidemia which is characterized by decreased levels of high-density lipoprotein cholesterol (HDL-C) is common [10, 11]. Likewise, low HDL-C is common in T2DM patients [12]. Apolipoprotein A-I (apo A-I) makes up the major apolipoprotein in HDL-C and reflects antiatherogenic properties [13]. Waldman et al. [14] observed that lower HDL-C/apo A-I predicted long-term glycemic deterioration in T2DM. However, whether HDL-C/apo A-I is associated with NAFLD remains unclear.

In this study, we conducted a cross-sectional survey to analyze the association between HDL-C/apo A-I and NAFLD. We also explored whether HDL-C/apo A-I is associated with the risk of NAFLD in lean subjects (BMI < 23 kg/m^2^) and with the degree of hepatic fibrosis assessed by FIB-4.

## 2. Materials and Methods

### 2.1. Study Population

A total sample of 9025 participants (6231 men and 2794 women) were drawn from adults who took their annual health examination at Zhenhai Lianhua Hospital, Ningbo, during 2017. Participants were viewed as disqualified for this study if they had a history of other etiologies of chronic liver disease including viral hepatitis or with alcohol consumption greater than 210 g/week for males and 140 g/week for females [1]. The study was approved by the Hospital Ethics Committee.

### 2.2. Clinical Examinations

Clinical examinations were carried out according to previous procedures [9, 15–17]. Standing height and body weight were measured in standard procedures. Waist circumference was measured when the subjects were standing up with the abdomen relaxed using an inelastic tape. Systolic and diastolic blood pressures were recorded by using a sphygmomanometer with the arm supported at the heart level.

Venous blood samples were collected after the overnight-fasting period. The biochemical markers, including liver enzymes, serum lipids, glucose, and uric acid, were measured as described by using an autoanalyzer [18]. HDL-C/apo A-I was calculated as HDL-C (mmol/L) divided by apo A-I (mmol/L).

### 2.3. Diagnosis of NAFLD, Metabolic Syndrome, and Advanced Hepatic Fibrosis

NAFLD was diagnosed by hepatic ultrasound according to the guidance supported by the Chinese Liver Disease Association [1]. The experienced ultrasonographists who were blind to the study design and clinical laboratory data carried out the ultrasound examination using a 3.5 MHz transducer (Nemio 20, Toshiba, Japan).

Metabolic syndrome (MetS) was diagnosed according to the definition suggested by the Asia-Pacific Working Party on NAFLD [19], with the exception of BMI, which was based on the literature that recommended BMI ≥ 23 kg/m^2^ in Asians as the definition of overweight [20]. MetS was diagnosed if any three or more of the following were met: (i) central obesity: BMI ≥ 23 kg/m^2^ for both genders and/or waist circumference ≥90 cm for males and ≥80 cm for females; (ii) hypertriglyceridemia: triglycerides ≥1.7 mmol/L; (iii) low high-density lipoprotein cholesterol (HDL-C): HDL-C <1.03 mmol/L for males and <1.29 mmol/L for females; (iv) elevated blood pressure: systolic blood pressure ≥130 mmHg or diastolic blood pressure ≥85 mmHg; and (v) elevated fasting blood glucose: fasting blood glucose ≥5.6 mmol/L or previously diagnosed type 2 diabetes.

According to the guidance of the American Association for the Study of Liver Diseases [4], advanced hepatic fibrosis is defined as FIB-4 >3.25.

### 2.4. Statistical Analysis

Continuous variables were presented as the mean and standard deviation (SD) when normally distributed or as the median and interquartile range (IQR) if skewed. Student's *t* test, the Mann–Whitney U test, and the chi-squared (*χ*^2^) test were used for comparisons of differences between groups. Correlations between HDL-C/apo A-I and NAFLD-related metabolic features were calculated by Pearson's or Spearman's analysis. Univariate and multivariate logistic regression analysis (backward: Wald; cutoff for entry: 0.05, for removal: 0.10) was applied to assess the risk factors for NAFLD. All statistical analyses were performed using SPSS 20.0 software for Windows (SPSS Inc., Chicago, IL). *P* < 0.05 (2-tailed test) was considered statistically significant.

## 3. Results

### 3.1. Clinical Characteristics of the Subjects with or without NAFLD

Of the 9025 enrolled subjects, 3038 (33.7%) fulfilled the diagnostic criteria of NAFLD, and the prevalence in men and women was 38.3% and 23.2%, respectively. Clinical characteristics of the participants according to NAFLD status were presented in Table 1. NAFLD patients were older, male predominant, and had higher body mass index, waist circumference, systolic and diastolic blood pressure, alanine aminotransferase, aspartate aminotransferase, *γ*-glutamyl transferase, triglyceride, total cholesterol, low-density lipoprotein cholesterol, fasting plasma glucose, serum uric acid, and glycated hemoglobin A1c, but had lower levels of serum HDL-C and apo A-I than NAFLD-free subjects. These results showed that metabolic parameters were less favorable in NAFLD patients compared with the controls. Notably, we found NAFLD patients had markedly lower HDL-C/apo A-I than controls (1.00 (0.88–1.13) versus 1.02 (0.91–1.13), *P* < 0.001; Table 1).

Our subgroup analysis found that HDL-C/apo A-I was significantly lower in lean NAFLD patients (BMI < 23 kg/m^2^) than that in lean controls (1.00 (0.87–1.11) versus 1.01 (0.92–1.13), *P* < 0.001). Regarding to hepatic fibrosis assessed by FIB-4, we found that NAFLD patients with advanced fibrosis (FIB-4 >3.25) had significantly lower HDL-C/apo A-I than controls (0.95 (0.81–1.11) versus 1.02 (0.91–1.13), *P* < 0.001). These results suggested that HDL-C/apo A-I may be also associated with lean NAFLD and NAFLD-related advanced fibrosis.

### 3.2. Association between HDL-C/apo A-I and Prevalence of NAFLD

We classified all the subjects into two groups according to the median level of HDL-C/apo A-I (1.01). We found that subjects with HDL-C/apo A-I ≥1.01 had significantly higher prevalence of NAFLD than those with HDL-C/apo A-I <1.01 (35.3% versus 32.0%, *χ*^2^ = 10.988, *P* < 0.001). Besides, we observed a significantly higher prevalence of main components of MetS including hypertriglyceridemia, hypertension, and low HDL-C in subjects with HDL-C/apo A-I ≥1.01 compared with those with HDL-C/apo A-I <1.01 (Table 2).

### 3.3. Association between HDL-C/apo A-I and NAFLD-Related Metabolic Parameters

To better understand the association between HDL-C/apo A-I and NAFLD, we further analyzed the correlation between HDL-C/apo A-I and metabolic features. Spearman's analysis showed that HDL-C/apo A-I was significantly and negatively associated with systolic blood pressure, alanine aminotransferase, aspartate aminotransferase, *γ*-glutamyl transferase, triglyceride, fasting plasma glucose, and glycated hemoglobin A1c (Table 3). These results indirectly support the association between HDL-C/apo A-I and NAFLD.

### 3.4. Association between HDL-C/apo A-I and Risk of NAFLD

We performed univariate and multivariate logistic regression analysis to explore whether HDL-C/apo A-I is independently associated with the risk of NAFLD. In the univariate model, HDL-C/apo A-I was significantly associated with a decreased risk of NAFLD (OR: 0.551, 95% CI: 0.433–0.701; *P* < 0.001) (Table 4). In the multivariate model, after adjusting for body mass index, diastolic blood pressure, alanine aminotransferase, *γ*-glutamyl transferase, total cholesterol, uric acid, and glycated hemoglobin A1c, HDL-C/apo A-I remained significantly associated with the risk of NAFLD (OR: 0.353, 95% CI: 0.257–0.486, *P* < 0.001) (Table 5). These results further support a negative association between HDL-C/apo A-I and NAFLD.

## 4. Discussion

2In this study, we found that HDL-C/apo A-I was negatively associated with NAFLD. First, HDL-C/apo A-I was significantly decreased in NAFLD patients, as well as in lean NAFLD and in patients with NAFLD-related advanced fibrosis. Second, HDL-C/apo A-I was negatively associated with the prevalence of NAFLD. Third, HDL-C/apo A-I was negatively and independently associated with the risk of NAFLD.

The serum levels of HDL-C have been shown to be negatively associated with cardiovascular disease (CVD) risks. Therefore, many clinical trials aimed at increasing HDL-C levels have been carried out to see whether this strategy could benefit decreasing CVD burden. However, neither cholesterol ester transfer protein inhibitors nor niacin as powerful HDL-C-level-increasing drugs is effective for decreasing cardiovascular event rates [21, 22].

Notably, HDL is a heterogenous particle, varying in size, composition, and function, and thus, HDL particles rather than the levels of HDL-C are better biomarkers of the role of HDL. As predicted by Shen [23], Mazer et al. [24] observed the quasilinear relationship between HDL size and HDL-C/apo A-I among the Women's Health Study Data. HDL-C/apo A-I, an indicator of HDL particle size and cholesterol content, has been explored in several studies regarding its association of CVD as follows. Sung et al. [25] observed that increased HDL-C/apo A-I was related to the increased risk of CVD, cancer, and all-cause mortality in a Korean occupational cohort of 263340 people. In a subset of 12031 people from the same cohort, coronary artery calcium scores (marker of preclinical atherosclerosis) were found to be positively associated with HDL-C/apo A-I [26]. In 2566 statin-treated patients who underwent intravascular ultrasound to evaluated atheroma burden, elevated levels of HDL-C/apo A-I rather than increases in HDL-C or apo A-I were associated with less progression of coronary atherosclerosis [27]. Another study performed in 2529 Chinese population found a U-shaped association of HDL-C/apo A-I and peak cardiac troponin I within 24 hours after percutaneous coronary intervention [28]. These studies suggest that cholesterol-rich and apo A-I-poor HDL particle (high HDL-C/apo A-I ratio) is associated with an increased risk of preclinical atherosclerosis.

In contrast, regarding long-term progression of hyperglycemia in T2DM patients, lower HDL-C/apo A-I was found to predict earlier and greater uptake of pharmacologic glucose control for those who only need lifestyle intervention at baseline [14]. Similarly, Abbasi et al. [29] found that higher HDL-C/apo A-I was significantly related to a lower risk of incident T2DM. These contradictory observations may be the result of different roles of HDL in the pathogenesis of CVD and T2DM, or different ethics. Furthermore, NAFLD is believed to be closely related to lifestyle-related diseases such as CVD and T2DM. To the best of our knowledge, this is the first study to reveal that HDL-C/apo A-I levels were significantly and independently associated with NAFLD.

Various lipoproteins and apolipoproteins are closely related to the risk of NAFLD. A cross-sectional study involving 9162 individuals in South Korea found that compared with the first quartile of apolipoprotein B to A-I ratio (apo B/apo A-I), the fourth quartile of apo B/A-I was associated with 1.359-fold risk of NAFLD [30]. Apo B/apo A-I was also positively associated with carotid intima media thickness in NAFLD patients [31]. Besides, elevated serum apolipoprotein E, very-low-density lipoprotein, and low-density lipoprotein were independently associated with NAFLD [32, 33].

The underlying mechanism linking HDL-C/apo A-I to NAFLD remains to be elucidated. There are several possible explanations. First, systemic low-grade inflammation has played a central role in the pathogenesis of NAFLD. HDL particles display anti-inflammatory functions via inhibiting production of multiple proinflammatory cytokines and chemokines and decreasing adhesion molecule expression [34]. Regarding T2DM, reduced HDL-C/apo A-I is thought to promote islet cell stress via association of systemic low-grade inflammation [14]. Second, oxidative stress is a well-known risk factor in the progression of NAFLD [35]. HDL particles represent to accept oxidized lipids and to inhibit LDL oxidation, especially in the process of antiatherosclerosis [34]. Third, HDL particles promote cholesterol efflux from macrophage foam cells, and drugs aimed at promoting reverse cholesterol transport showed protective function in liver steatosis [36]. Fourth, insulin resistance is the main component of NAFLD, and an inverse association was found between HDL particle size and insulin resistance. To be more specific, insulin resistance progression was related to an increase of small HDL particles and a decrease of large HDL particles measured by nuclear magnetic resonance [37].

Our study has several limitations. First, NAFLD was diagnosed by hepatic ultrasonography which is not sensitive for mild hepatic steatosis. Second, the determination of causality is not clear in the cross-sectional design. Third, with regard to the underlying mechanisms of association between HDL-C/apo A-I and NAFLD, we did not assess markers of systemic low-grade inflammation such as high-sensitivity C response protein, interleukin-6. Similarly, insulin resistance was not examined. Fourth, in this study, we assessed the degree of hepatic fibrosis by FIB-4, which is a cost-effective and noninvasive diagnostic score for assessing fibrosis in NAFLD patients [4]. But, its ability to exclude advanced fibrosis is more useful than identifying it [38]. Although the diagnostic utility has been shown to be accurate in several clinical settings [39], its diagnostic ability is limited in individuals aged ≤35 years or aged ≥65 years [40].

In conclusion, our large cross-sectional study suggests that HDL-C/apo A-I, a useful surrogate biomarker for HDL particle size, is inversely associated with NAFLD.

## Figures and Tables

**Table 1:** Comparison of clinical characteristics between the participants with and without NAFLD.

Variables	With NAFLD (*n* = 3038)	Without NAFLD (*n* = 5987)	*Z* value	*P* value
Age (year)	52 (43–63)	49 (39–62)	−7.577	<0.001
Gender (male/female, *n*)	2389/649	3842/2145	197.283^†^	<0.001
Body mass index (kg/m^2^)	25.10 (23.53–27.00)	22.00 (20.40–23.70)	−49.467	<0.001
Waist circumference (cm)	89 (85–94)	80 (74–86)	−44.467	<0.001
Systolic blood pressure (mmHg)	132 (122–142)	124 (113–135)	−22.649	<0.001
Diastolic blood pressure (mmHg)	80 (72–87)	73 (66–81)	−24.292	<0.001
Alanine aminotransferase (U/L)	26 (19–40)	17 (13–23)	−38.158	<0.001
Aspartate aminotransferase (U/L)	23 (19–29)	20 (17–25)	−21.456	<0.001
*γ*-Glutamyl transferase (U/L)	32 (23–50)	20 (15–28)	−37.811	<0.001
Triglyceride (mmol/L)	1.83 (1.34–2.56)	1.13 (0.85–1.56)	−40.646	<0.001
Total cholesterol (mmol/L)	4.99 (4.38–5.65)	4.71 (4.14–5.35)	−12.828	<0.001
HDL-C (mmol/L)	1.19 (1.03–1.35)	1.31 (1.15–1.49)	−21.573	<0.001
LDL-C (mmol/L)	2.82 (2.34–3.33)	2.74 (2.31–3.22)	−4.408	<0.001
Apo A-I (mmol/L)	1.18 (1.05–1.32)	1.27 (1.12–1.46)	−18.130	<0.001
Fasting plasma glucose (mmol/L)	5.42 (5.05–5.97)	5.18 (4.91–5.54)	−18.705	<0.001
Serum uric acid (*μ*mol/L)	382 (329–434)	328 (276–380)	−28.709	<0.001
HbA1c (%)	5.50 (5.20–5.90)	5.30 (5.10–5.60)	−21.146	<0.001
HDL-C/apo A-I	1.00 (0.88–1.13)	1.02 (0.91–1.13)	−5.207	<0.001

Data are presented as median (IQR) due to skewed distribution. ^†^*χ*^2^ value; apo A-I, apolipoprotein A-I; HbA1c, glycated hemoglobin A1c; HDL-C, high-density lipoprotein cholesterol; HDL-C/apo A-I, high-density lipoprotein cholesterol to apolipoprotein A-I ratio; LDL-C, low-density lipoprotein cholesterol; NAFLD, nonalcoholic fatty liver disease.

**Table 2:** Prevalence rate of NAFLD and MetS components in participants with HDL-C/apo A-I greater or less than 1.01.

	Participants with HDL-C/apo A-I ≥1.01	Participants with HDL-C/apo A-I <1.01	*P* value
NAFLD (%)	35.3	32.0	<0.001
Hypertriglyceridemia (%)	39.9	25.0	<0.001
Hypertension (%)	48.5	44.2	<0.001
Low HDL-C (%)	38.0	9.7	<0.001

HDL-C: high-density lipoprotein cholesterol; HDL-C/apo A-I: high-density lipoprotein cholesterol to apolipoprotein A-I ratio; NAFLD: nonalcoholic fatty liver disease.

**Table 3:** Correlations between HDL-C/apo A-I and metabolic parameters.

	SBP	ALT	AST	GGT	TG	FPG	HbA1c
*r* value	−0.062	−0.063	−0.123	−0.078	−0.200	−0.057	−0.029
*P* value	<0.001	<0.001	<0.001	<0.001	<0.001	<0.001	0.005

ALT, alanine aminotransferase; AST, aspartate aminotransferase; FPG, fasting plasma glucose; GGT, *γ*-glutamyl transferase; HbA1c, glycated hemoglobin A1c; SBP, systolic blood pressure; TG, triglyceride.

**Table 4:** Univariable analysis for factors associated with NAFLD.

Variables	OR	95% CI	*P* value
Gender (male/female)	2.055	1.857–2.275	<0.001
Age (year)	1.011	1.008–1.013	<0.001
Body mass index (kg/m^2^)	1.635	1.598–1.674	<0.001
Waist circumference (cm)	1.149	1.141–1.157	<0.001
Systolic blood pressure (mmHg)	1.030	1.027–1.032	<0.001
Diastolic blood pressure (mmHg)	1.053	1.048–1.057	<0.001
Alanine aminotransferase (U/L)	1.056	1.052–1.060	<0.001
Aspartate aminotransferase (U/L)	1.050	1.045–1.056	<0.001
*γ*-Glutamyl transferase (U/L)	1.020	1.018–1.022	<0.001
Triglyceride (mmol/L)	2.770	2.600–2.951	<0.001
Total cholesterol (mmol/L)	1.339	1.279–1.402	<0.001
LDL-C (mmol/L)	1.145	1.078–1.216	<0.001
Fasting plasma glucose (mmol/L)	1.478	1.408–1.552	<0.001
Serum uric acid (*μ*mol/L)	1.008	1.008–1.009	<0.001
HbA1c (%)	2.062	1.902–2.235	<0.001
HDL-C/apo A-I	0.551	0.433–0.701	<0.001

HbA1c, glycated hemoglobin A1c; HDL-C/apo A-I, high-density lipoprotein cholesterol to apolipoprotein A-I ratio; LDL-C, low-density lipoprotein cholesterol.

**Table 5:** Multivariable analysis for factors associated with NAFLD.

Variables	OR	95% CI	*P* value
Body mass index (kg/m^2^)	1.504	1.467–1.541	<0.001
Diastolic blood pressure (mmHg)	1.022	1.016–1.027	<0.001
Alanine aminotransferase (U/L)	1.032	1.027–1.036	<0.001
*γ*-Glutamyl transferase (U/L)	1.002	1.000–1.004	0.015
Total cholesterol (mmol/L)	1.297	1.219–1.381	<0.001
HbA1c (%)	1.628	1.494–1.774	<0.001
Serum uric acid (*μ*mol/L)	1.004	1.003–1.004	<0.001
HDL-C/apo A-I	0.353	0.257–0.486	<0.001

HbA1c, glycated hemoglobin A1c; HDL-C/apo A-I, high-density lipoprotein cholesterol to apolipoprotein A-I ratio.

## Data Availability

The data used to support the findings of this study are available from the corresponding author upon request.
